# Remediation of hexavalent chromium contaminated water through zero-valent iron nanoparticles and effects on tomato plant growth performance

**DOI:** 10.1038/s41598-020-58639-7

**Published:** 2020-02-05

**Authors:** Elisa Brasili, Irene Bavasso, Valerio Petruccelli, Giorgio Vilardi, Alessio Valletta, Chiara Dal Bosco, Alessandra Gentili, Gabriella Pasqua, Luca Di Palma

**Affiliations:** 1 Sapienza University of Rome, Department of Environmental Biology, Rome, 000185 Italy; 2 Sapienza University of Rome, Department of Chemical Engineering Materials Environment, Rome, 00185 Italy; 3 Sapienza University of Rome, Department of Chemistry, Rome, 00185 Italy

**Keywords:** Plant stress responses, Nanoscience and technology

## Abstract

Contaminated water with hexavalent chromium Cr(VI) is a serious environmental problem. This study aimed to evaluate the Cr(VI) removal by zero valent iron nanoparticles (nZVI) reduction process and the impact of Cr(VI), nZVI and combined treatment with nZVI and Cr(VI) on tomato growth performance. To evaluate the Cr(VI) toxic effect on germination capability, seeds were exposed to increasing Cr(VI) concentrations up to 1000 mg L^−1^. The inhibition of seed germination and the decrease of hypocotyl and root length started from Cr(VI) 5 mg L^−1^. Under treatment with Cr(VI) + nZVI 5 mg L^−1^, seed germination, hypocotyl and root length resulted significantly higher compared to Cr(VI) 5 mg L^−1^ treatment. The impact of only nZVI was investigated on chlorophyll and carotenoid in leaves; iron levels in leaves, roots, fruits and soil; carotenoid, fat-soluble vitamin and nicotianamine in mature fruits. A significant increase of leaf chlorophyll and carotenoids was observed after nZVI 5 mg L^−1^ treatment compared to controls. No significant variations were observed in carotenoids, fat-soluble vitamins and nicotianamine levels after treatment with nZVI 5 mg L^−1^ in mature fruits. For their ability to reduce Cr(VI) and to stimulate tomato growth, nZVI might to be considered as alternative for remediation purposes.

## Introduction

According to the Agency for Toxic Substances and Disease Registry and the International Agency for Research on Cancer, chromium (Cr) has been ranked 7^th^ among the top 20 hazardous substances and no.1 carcinogen^1,2^. Cr occurs in different oxidation states (−2 to + 6), but hexavalent chromate Cr(VI) and trivalent chromite Cr(III) forms are the most common and stable in the natural environment^3^. Compared to Cr(III), Cr(VI) is highly mobile in soil, extremely toxic to living organisms with mutagenic, carcinogenic and teratogenic potential^4^. On the other hand, Cr(III) is significantly less toxic and serves as an essential element in trace amounts. Environmental contamination of Cr(VI) is gaining increasing consideration worldwide due to its high levels in the water and soil deriving from natural and anthropogenic activities including industrial applications such as metallurgical, refractories and chemicals^3^. The exposure to Cr(VI) can cause several serious diseases in nervous, kidney, hematopoietic and gastrointestinal systems of humans^5–7^. Cr(VI) is also a toxic heavy metal for plants and it is harmful to their development and metabolism, interfering with plant growth, nutrient uptake and photosynthesis, inducing enhanced generation of reactive oxygen species, causing lipid peroxidation and altering the antioxidant activities^8^. It is also important to stress that Cr uptake by crops and accumulation in edible plant parts organs with serious risks for consumer health^5,9^.

In order to remove Cr(VI) from soil and water and eventually reuse the reclaimed water for irrigation purposes, several methods have been researched and reviewed extensively. These include chemical reduction to Cr(III), solvent extraction, chelation and adsorption, among others^10–12^. In recent years, zero valent iron nanoparticles (nZVI) have attracted a great deal of attention due to their efficiency in removal of different types of contaminants from aqueous solutions. nZVI are characterized by a core-shell structure, with the core made of metallic iron and the shell constituted of oxide and hydroxydes of Fe(II) and Fe(III)^13^. Relatively little is known about the potential of using nanoparticles for the removal of Cr(VI) from aqueous systems. In a recent study, Zhao *et al*.^14^ described a novel amino-functionalized vermiculite (AVT)-supported nanoscale zero-valent iron (AVT-nZVI) for Cr(VI) removal from simulated electroplating rinse wastewater. Wang *et al*.^15^ demonstrated that zero-valent iron (Fe^0^) nanoparticles (NPs) with 4% bentonite were efficient for the treatment of Cr(VI)-containing wastewaters. The feasibility of Cr(VI) removal through nZVI has been also verified in our previous works when present alone^16,17^ and in co-presence with other pollutants (nitrates)^18^. The good results obtained by this process and the need to operate with excess when not only Cr(VI) is present as a contaminant, made necessary to carry out a study on the effects of the residual nanoparticles in the water. This is linked with the aim of this work which represents an investigation of the effects of nanoparticles on plant growth. Indeed, only a limited amount of studies has been conducted to evaluate the phytotoxicity of nZVI despite of the fact that exposure to plants is due to the intentional injection into the soils^19,20^ and to the best of our knowledge, there are not report regarding the use of nZVI on tomato plants.

The present research deals with a comprehensive study of Cr(VI) removal from contaminated water by nZVI reduction process and of the impact of nZVI when present alone and in co-presence with Cr(VI) on *Solanum lycopersicum* growth performance. In particular, the toxic effect of different concentrations of Cr(VI) has been evaluated on tomato seed germination and seedling development. The effects of the water treated with nZVI have been also studied on tomato growth performance in terms of chlorophyll and carotenoid content in the leaves. Moreover, a chemical profile of the metabolites produced in the mature fruits, both in terms of carotenoids, fat-soluble vitamins and nicotianamine (NA) content has been carried out to investigate the impact of nZVI on plant metabolism.

## Results and Discussion

### Kinetic study

**Figure 1:**
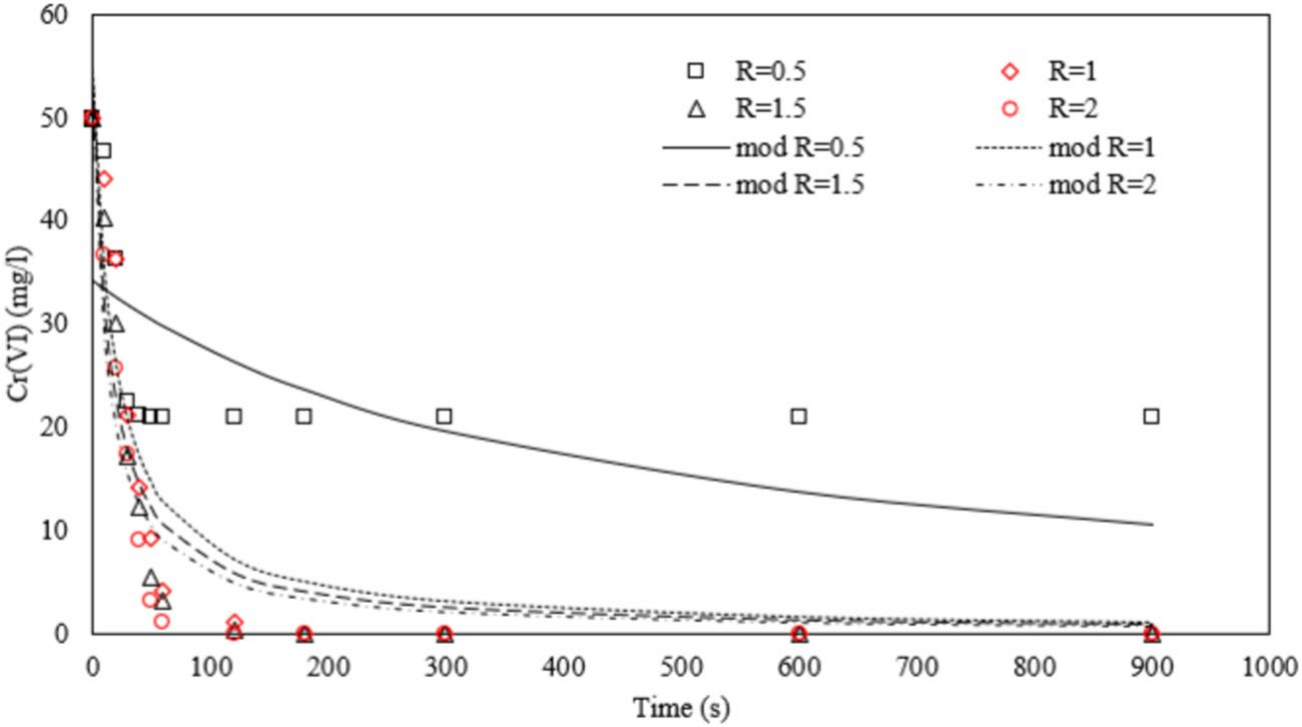
Kinetic data modelling of Cr(VI) removal by nZVI (stirring intensity 500 rpm, temperature = 25 °C, R = nZVI/Cr(VI) molar ratio).

As shown in Fig. 1, the kinetic model proposed in a previous work^11^, proved to be able to describe the results obtained at nZVI/Cr(VI) molar ratio (R) ≥ 1, while when the particles were added below the stoichiometric molar ratio, the asymptotic behavior of the experimental data trend was not well fitted. In particular, when nanoparticles were added a similar initial trend was observed for all the investigated R, but at R = 0.5, due to the low amount of nZVI in solution, reaction stopped at a removal of about 50%. Conversely, when a sufficient dosage was provided (starting from the stoichiometric amount, R = 1), a quantitative removal was obtained within 120 min of treatment. In the absence of other competitive species for nZVI oxidation a stoichiometric dosage of nZVI with respect to the concentration of Cr(VI) is therefore recommended. Any excess of nanoparticles can undergo a quick oxidation by oxygen in water: their persistence and the effect on plant growth when treated water is used for irrigation is therefore worth of investigation.

The apparent kinetic constant obtained from the kinetic data modelling slightly varied with R, in detail the k varied from 8 × 10^−4^ mg^1−n^/l^1−n^ s up to 0.0015 mg^1−n^/l^1−n^ s for R = 0.5 up to R = 2 whereas the reaction order n, varied in the range 1.9–2.1 passing from R = 0.5 up to R = 2. The determination coefficient R^2^ was 0.8 for R = 0.5, whereas was in the range 0.93–0.95 for R = 1–2.

### Effect of Cr(VI) on tomato seed germination

Seed germination is a crucial process that influences crop yield and quality. The increase of Cr(VI) concentration from 5 to 1000 mg L^−1^ adversely affected the germination gradually decreasing the tomato seed germination percentage (Table 1).

**Table 1:** Seed germination percentage at 3, 6, and 10 days after treatment with different Cr(VI) and nZVI concentrations.

Treatment	Time		
	3 days	6 days	10 days
Control	82%	94%	100%
Cr(VI) 5 mg L^−1^	56%	78%	82%
Cr(VI) + nZVI 5 mg L^−1^	82%	92%	100%
nZVI 5 mg L^−1^	100%	100%	100%
Cr(VI) 50 mg L^−1^	46%	74%	78%
Cr(VI) + nZVI 50 mg L^−1^	76%	96%	100%
nZVI 50 mg L^−1^	88%	100%	100%
Cr(VI) 100 mg L^−1^	40%	70%	76%
Cr(VI) + nZVI 100 mg L^−1^	56%	80%	96%
nZVI 100 mg L^−1^	72%	88%	96%
Cr(VI) 1000 mg L^−1^	6%	20%	26%
Cr(VI) + nZVI 1000 mg L^−1^	0%	4%	4%
nZVI 1000 mg L^−1^	0%	4%	4%

Germination in tomato seed exposed to different Cr(VI) and nZVI concentrations at 3, 6, and 10 days. Each sample consists of a dish with 50 seeds. Data are tabulated as % germination. Differences between means were compared using Chi-Squared test. P < 0.005 was considered to indicate a statistically significant difference.

The negative effect of Cr(VI) on seed germination was previously explored in different plants of agronomic interest. Rout *et al*.^21^ demonstrated that the treatment with 200 *μ*M Cr(VI) reduced by 25% germination of *Echinochloa colona* L. seeds. The presence of Cr(VI) at high concentrations in the soil reduced to 48% seed germination in *Phaseolus vulgaris* L^22^. Decreased seed germination with increasing concentration of chromium ions was also observed for cowpea *Vigna sinensis* L.^23^, melon (*Cucumis melo* L.)^24^, and wheat (*Triticum aestivum* L.)^25^. As it has been proposed, chromium could activate proteases or inhibit amylase activity with the subsequent decreased transport of carbohydrates to the germ leading to the seed death or delayed seed germination^26^.

In this study, a significant increase of seed germination with increasing culture time (3,6 and 10 days) was also observed in all experimental groups (Table 1). In addition, after the transfer of seeds to deionized H_2_O, they still germinated nearly 100% (data not shown).

Once Cr(VI) overcome biophysical barriers, seeds could initiate several cellular defense mechanisms to nullify and attenuate the adverse effects of chromium, accumulating, storing and immobilizing the heavy metal by binding them with amino acids, proteins or peptides. Tong *et al*.^27^ reported that plant firstly tries to prevent metal transport across the plasma membrane by binding or modification of metal ions. Secondly, metal ions which enter the plant body are detoxified through inactivation or converted into less toxic form.

### Effect of nZVI on tomato seed germination

Chi-Square test revealed significant statistical differences between germination percentage at all experimental times and treatments (Table 1). In particular, after treatment with nZVI 5 mg L^−1^, 100% seed germination was observed at all culture times. A positive effect on seed germination was observed also after treatment with nZVI 50 mg L^−1^. A recent study of Li *et al*.^28^ showed that exposure of peanut seeds to low concentration of nZVI (40–80 µmol L^−1^ nZVI) can promote the germination rate. The authors have proposed that the nanoparticles can penetrate the plant seed coat to support water uptake. In support of this evidence, Guha *et al*.^29^ found that rice seeds treated with 20 mg L^−1^ nZVI showed the fastest water uptake, increased amylase and protease enzyme activities and seedling vigour. This mechanism could also be applied to explain the findings of this work. nZVI at 5 and 50 mg L^−1^ may penetrate the tomato seed coats increasing the water uptake to stimulate seed germination.

However, the exposure of tomato seeds to nZVI at 100 and 1000 mg L^−1^ negatively affected the seed germination. This inhibitory effect on germination was previously observed in other plant species such as cattail (*Thyfa latifolia*) and hybrid poplars (*Populus deltoids* x *Populus nigra*) treated with high concentrations of nZVI (>200 mg L^−1^)^19^. It can be proposed that high concentrations of nZVI forming a coating on seed surface could alter membrane pores and interfere with the water and nutrient uptake process. On the other hands, Ma *et al*.^19^ observed the formation of black coating on root surface on *Thyfa latifolia* and *Populus deltoids x Populus nigra*, after treatment with nZVI (>200 mg L^−1^).

Seed germination percentage was always higher after nZVI treatment compared to Cr(VI) + nZVI and Cr(VI) at all tested concentrations. In addition, after treatment with Cr(VI) + nZVI, the seed germination was significantly higher compared to Cr(VI) treatment at all tested concentrations.

Although the ability of nZVI to reduce Cr(VI) and improve tomato seed germination at 5 and 50 mg L^−1^, the improving of germination after nZVI + Cr(VI) treatment cannot be attributed only to reduction of Cr(VI) but the presence of nZVI could have contributed to the improvement in germination, compensating the negative effect of Cr(VI).

### Effect of Cr(VI) on tomato seedling development

**Figure 2:**
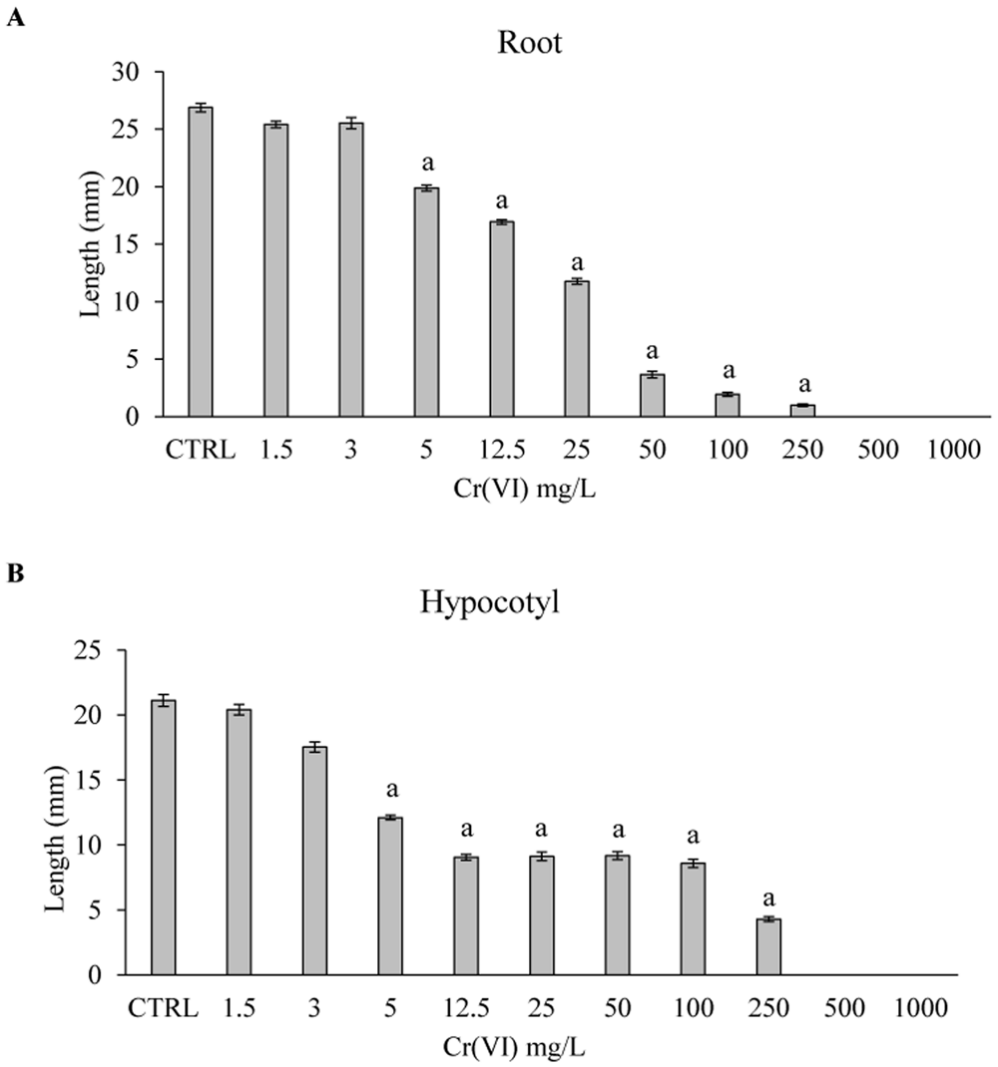
Length of root (**A**) and hypocotyl (**B**) in tomato seedlings after 7 days of treatment with different concentrations of Cr(VI). Data are expressed as the mean (n = 10) ± standard error. All experiments were performed in triplicate. The statistical analysis of differences was performed using ANOVA followed by Holm-Sidak Test. P < 0.05 was considered to indicate a statistically significant difference. Letter a indicates significant difference compared to the control group.

Figure 2A,B illustrates that the length of root and hypocotyl was inversely related with increase of Cr(VI) concentration showing a significant toxic effect of Cr(VI) on 7 day-old seedlings. Inhibition of seedling growth occurred starting from Cr(VI) 5 mg L^−1^ and gradually increased with increasing of Cr(VI) concentration. Tomato seedling growth was completely inhibited by Cr(VI) 500 and 1000 mg L^−1^. Among other toxic effects of chromium, the inhibition of root growth was widely observed. Inhibitory effects of chromium on the root elongation were found in *Vigna radiata* and *Glicine max*^30,31^. Daud *et al*.^32^ reported that chromium caused a significant reduction in cotton root/shoot length, number of secondary roots, and root fresh and dry biomasses at 100 *μ*M. The root growth defects under exposure to high levels of heavy metals can be caused by inhibition of root cell division and/or reduction of cell proliferation^33^.

**Figure 3:**
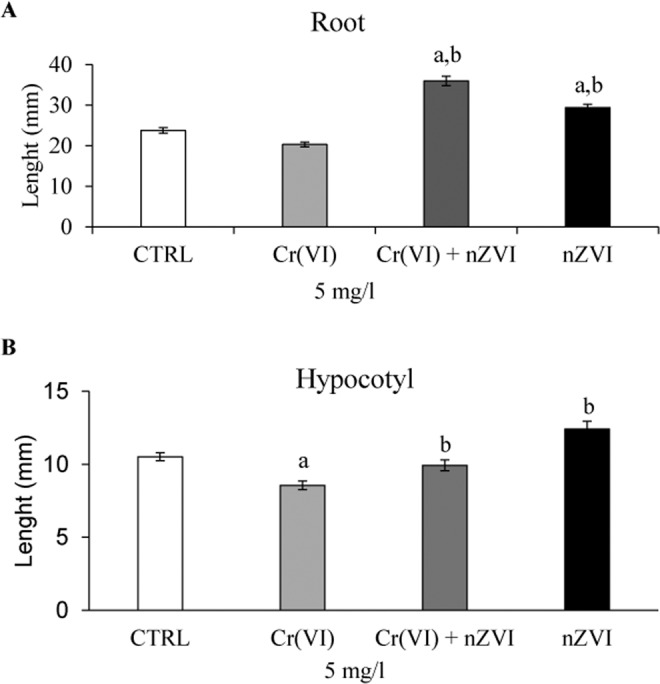
Length of root (**A**) and hypocotyl (**B**) in tomato seedlings after 7 days of treatment with Cr(VI) 5 mg L^−1^, nZVI 5 mg L^−1^, and Cr(VI) + nZVI 5 mg L^−1^. Data are expressed as the mean (n = 10) ± standard error. All experiments were performed in triplicate. The statistical analysis of differences was performed using ANOVA followed by Holm-Sidak Test. P < 0.05 was considered to indicate a statistically significant difference. Letter a indicates significant difference compared to the control group (*p* ≤ 0.05); letter b indicates significant difference compared to Cr(VI) 5 mg L^−1^.

Chromium severely affected tomato seedling development. As showed in Fig. 3, a significant decrease of hypocotyl (**B**) length after Cr(VI) 5 mg L^−1^ treatment compared to control plants was observed.

### Effect of nZVI and Cr(VI) + nZVI on tomato seedling development

The treatment with nZVI 5 mg L^−1^ significantly increased the length of both seedling root and hypocotyl compared to Cr(VI) treatment (Fig. 3A,B). The root development significantly improved after nZVI 5 mg L^−1^ treatment also compared to the control group (Fig. 3A). These results are congruent with previous studies demonstrating that low doses of nZVI enhance plant growth. Guha *et al*.^29^ observed that the treatment with 20 mg mg L^−1^ nZVI enhanced growth of aromatic rice cultivar (*Oryza sativa* cv. Gobindabhog L.) increasing root (~ 3.3 fold) and shoot (~ 1.52 fold) length, biomass and photosynthetic pigment content^30^. A recent study of Kim *et al*.^20^ showed that exposure of *Arabidopsis thaliana* to 500 mg L^−1^ nZVI can enhance root elongation because nZVI induces cell wall loosening. Ma *et al*.^19^ demonstrated that *Typha* plants treated with nZVI (<50 mg L^−1^) grew better than controls.

In our study, after the combined treatment Cr(VI) + nZVI 5 mg L^−1^ a significant increase of root and hypocotyl length was observed with respect of Cr(VI) 5 mg L^−1^ suggesting the ability of nZVI to reduce Cr(VI) restoring seedling growth (Fig. 3A,B).

Moreover, the root development significantly improved after Cr(VI) + nZVI 5 mg L^−1^ treatment compared to the control group (Fig. 3A) indicating that nZVI could have contributed to enhance seedling growth, compensating the negative effect of Cr(VI) and acting as potential nanofertilizer to increase plant vigour.

### Effect of nZVI on chlorophyll and carotenoid content in tomato leaves

**Figure 4:**
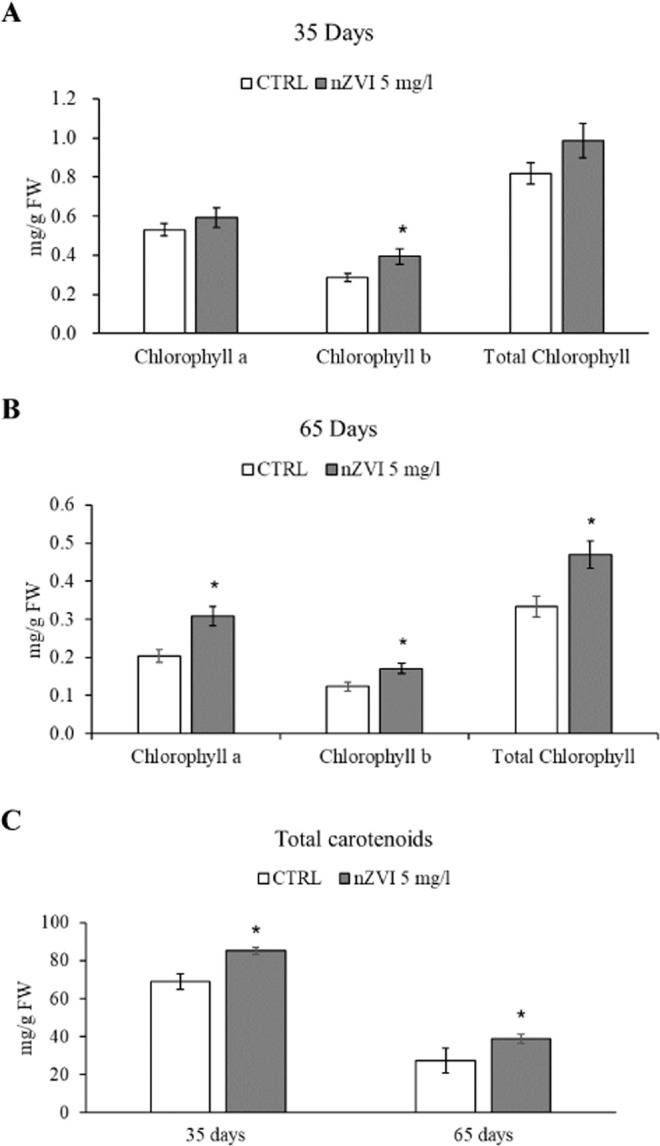
Leaf total chlorophyll at 35 days (**A**) and 65 days (**B**) of plant growth; Leaf total carotenoid at 35 days and 65 days (**C**) of plant growth. Data are expressed as the mean (n = 4) ± standard error. All experiments were performed in triplicate. The statistical analysis of differences was performed using t test of variance. P < 0.05 was considered to indicate a statistically significant difference. Asterisk indicates significant difference compared to control group.

After 35 days of treatment with nZVI 5 mg L^−1^, a significant increase of chlorophyll b was observed with respect to the control leaves. No significant differences were observed in total chlorophyll levels (Fig. 4A). At 65 days of plant growth, a significant increase of chlorophyll a and b as well as total chlorophyll was observed after nZVI 5 mg L^−1^ treatment compared to control plants (Fig. 4B). At 35 and 65 days, a significant increase of carotenoid level was observed in the leaves after treatment with nZVI 5 mg L^−1^ with respect to the control (Fig. 4C). These results suggest that low concentrations of nZVI (5 mg L^−1^) improved the photosynthetic pigment content of the tomato plants and no stress was induced due to the nZVI treatment. Similar results are previously obtained in rice seedlings treated with <80 mg L^−1^ nZVI^29^. Conversely, Wang *et al*.^34^ found that higher concentrations of nZVI (1000 mg L^−1^) in rice induced distinct signs of leaf chlorosis as chlorophyll and carotenoid content decreased markedly. In the present study, no significant variations were observed in number of nods and internods as well as number of flowers and plant height after treatment with nZVI 5 mg L^−1^ (data not shown). These findings demonstrate that nZVI at low concentration (5 mg L^−1^) can improve the efficiency of photosynthesis without any adverse effects on tomato plant development.

### Determination of iron level in leaves, roots, fruits and soil in different stages of plant development after nZVI treatment

After treatment with nZVI 5 mg L^−1^, iron concentrations in tomato roots were 2.56 and 8.29 mg/g DW at 35 and 65 days of plant development, respectively. It was previously found that the nanoparticles are deposited preferentially in tomato root hairs, root tip followed by nodal and middle zone of stem^35^.

In the present study, iron levels were detectable in the leaves (0.25 mg/g DW) only at 65 days of plant growth. Plant soil showed iron levels equal to 0.71 and 1.87 mg/g DW at 35 and 65 days of plant growth, respectively. Fe levels were not detected in leaves, roots, fruits and soil of the control plants. In addition, Fe was not found in mature fruits.

### Determination by LC-MS/MS of carotenoid, fat-soluble vitamin and nicotianamine content in the mature fruits after nZVI treatment

For the LC-MS analysis fruits at the same stage of ripening were collected. The medium number of fruits was 13 in control plants and 15 in treated plants. The medium weight of control fruits was 7.74 ± 2.11 g (SD) and 8.5 ± 1.9 (SD) for treated plants. No significant differences were observed between control and treated plants.

Carotenoid, fat-soluble vitamin and nicotianamine levels are listed in Table 2. No significant variations were observed among control and nZVI 5 mg L^−1^ samples, apart from β-carotene that significantly increased in mature fruits after treatment with nZVI 5 mg L^−1^. It is important to remember that carotenoids and vitamins are involved in a wide range of plant physiological processes, including growth, development, and responses to environmental stimuli. Among the most important carotenoids, β-carotene represents about 7% of the total carotenoid content^36^. Carotenoid content is also one of the most important quality traits of tomato fruit, with industrial, health, and nutritional properties. The amount of carotenoids is correlated to fruit quality; a greater carotenoid content make a more nutritious and tastier tomato^37^. Moreover, carotenoids are essential components of human diets that provide precursors for biosynthesis of vitamin A, a well-known carotenoid derivative with widespread biological functions^38^.

**Table 2:** Carotenoid, fat-soluble vitamin and nicotianamine levels in mature fruits after 180 days of nZVI 5 mg L^−1^ treatment.

Metabolite	Control (µg/g)	Treated (µg/g)	Test.t *p*-value
ɑ-tocopherol	4.37 ± 0.43	3.99 ± 0.23	NS
β-carotene	1.88 ± 0.22	2.79 ± 0.43	<0.05
phytoene	86.00 ± 4.04	94.36 ± 3.94	NS
phytofluene	43.21 ± 2.97	50.01 ± 3.43	NS
lutein	0.82 ± 0.04	0.97 ± 0.11	NS
zeaxanthin	9.66 ± 0.61	11.21 ± 1.15	NS
ζ-carotene	0.50 ± 0.14	0.50 ± 0.04	NS
β + γ tocopherol	7.01 ± 2.69	5.91 ± 0.81	NS
γ-carotene	0.88 ± 0.49	1.48 ± 0.22	NS
licopene	43.59 ± 14.74	57.08 ± 7.44	NS
nicotianamine	22.44 ± 1.96	25.00 ± 2.89	NS

Carotenoid, fat-soluble vitamin and nicotianamine levels in ripe fruits after 180 days of nZVI 5 mg L^−1^ treatment. Metabolites are expressed as the mean (n = 4) ± standard deviation (SD). The statistical analysis of differences was performed using t test of variance. P < 0.05 was considered to indicate a statistically significant difference. NS: not significant.

Our results demonstrated that nZVI 5 mg L^−1^ treatment increased β-carotene levels but not affected the main carotenoid and fat-soluble vitamin profile suggesting that the tomato fruit quality was not altered.

Differences between nicotinamine levels after treatment with nZVI 5 mg L^−1^ were not significant. The non-proteogenic amino acid nicotianamine (NA) is ubiquitous in higher plants and has been hypothesized to play a key role in the provision and distribution of iron and other metals in the plants^39^. It has been shown that NA synthesis is induced by high iron availability and its levels increase in tomato shoot apex and roots, under iron supply, indicating that it can play a role in protecting the cells against supraoptimal iron concentration (100 μM)^40^. Our observations showed that, even though Fe levels increased in roots after the application of nZVI 5 mg L^−1^, nicotianamine levels were not significantly increased suggesting that nZVI 5 mg L^−1^ not affected the Fe homeostasis in tomato plants.

## Conclusions

This study aimed to evaluate the Cr(VI) removal by nZVI reduction process and the impact of reclaimed water on *Solanum lycopersicum* sedd germination and seedling growth. In addition, the effect of low concentrations of nZVI (5 mg L^−1^) on tomato plant growth and metabolism was investigated. As the first of its kind, this work showed that the nZVI were able to convert Cr(VI) in Cr(III), a less toxic compound. Secondly, lower concentrations of nZVI (5 mg L^−1^) stimulated not only seed germination but also the root and hypocotyl growth of the tomato plantlets as well as the leaf chlorophyll and carotenoids levels. In this context, low concentrations of nZVI might be used to benefit the growth of tomato plants in agricultural settings. However high concentrations of nZVI (100 and 1000 mg L^−1^) adversely affected tomato seed germination. These result suggest that, when nanoremediation with nZVI is selected as the remediation method, it is important to consider both the remedial benefits and potential fate and toxicity of nZVI, and that the toxicity level is dependent upon plant species.

Interestingly, the combined treatment Cr(VI) + nZVI improved seedlings development compared to Cr(VI) treatment suggesting that nZVI could be used as strategy to reduce toxicity by Cr(VI) in remediation purposes. To the best of our knowledge, this is the first report to investigate the impact of nZVI on tomato, a species of agronomic interest.

## Material and Methods

### Chemicals and reagents

Methanol, ethanol, 2-propanol, hexane and acetonitrile (HPLC grade), as well as formic acid and butylated hydroxytoluene (BHT), were purchased from Sigma (St. Louis, MO, USA). Ultrapure water was produced by a Milli-Q Plus apparatus (Millipore, Bedford, MA, USA). Potassium hydroxide (KOH) and magnesium carbonate were obtained from Carlo Erba (Milan, Italy). The following standards were purchased from Aldrich-Fluka-Sigma Chemical (St. Louis, MO, USA): all-trans-β-carotene, all-trans-β-cryptoxanthin, all trans-zeaxanthin, retinol, β-tocopherol, δ-tocopherol, γ-tocopherol, α-tocopherol, all-trans-lutein, nicotianamine. Standards of α-tocotrienol, β-tocotrienol, δ-tocotrienol, and γ-tocotrienol were purchased from LGC Standards (Middlesex, U.K.). Standards of 15-cis-phytoene, all-trans-phytoene, all-trans-phytofluene, 15-cis-phytofluene, all-trans-ζ-carotene, all-trans-γ-carotene, all-trans-lycopene, and 5-cis-lycopene were bought from CaroteNature GmbH (Ostermundigen, Switzerland). All chemicals had a purity grade of >97%.

### Instrumentation

LC-MS/MS analyses were carried out using a micro HPLC series 200 (PerkinElmer, Norwalk, CT, U.S.A.) coupled to a 4000 Qtrap (AB SCIEX, Foster City, CA, U.S.A.) mass spectrometer. The Turbo V source was equipped with interchangeable atmospheric pressure chemical ionization (APCI) or electrospray ionization (ESI) probes. The Q1 and Q3 mass analyzers were preliminarly calibrated infusing a polypropylene glycol solution at 10 μL/min. A unit mass resolution was established by keeping full width at half maximum (FWHM) each mass-resolving quadrupole at 0.7 ± 0.1 u.

Q1−full scan spectra and product ion scan spectra of analytes were acquired by working in flow injection analysis (1−10 ng injected, 1 mL/min flow rate). Chromatograms were acquired in Multiple Reaction Monitoring (MRM) mode by picking two MRM ion currents per analyte. The most intense was used for the quantitative analysis, the second one for the analyte identification by comparing the ion ratio (i.e., the relative abundance of the selected MRM transitions) and the retention time with the values obtained for the authentic standard in the solvent. Tables S1 and S2 list LC−MRM parameters and linear regression parameters used for the quantitative analysis, respectively. Acquisition and elaboration of LC−MS/MS data were performed with Analyst 1.6.2 software (AB Sciex).

### Preparation and characterization of nanoparticle suspension

Preparation and characterization of nZVI particles were carried out as previously described by Vilardi *et al*.^17,41^. Briefly, nZVI particles were synthetized by borohydride reduction method, without the addition of any stabilizer. Iron sulfate hepta-hydrate (FeSO_4_·7H_2_O) solution (0.2 M) was reduced by sodium borohydride (NaBH_4_) solution (0.4 M) in a spinning Disk Reactor (SDR). All the used reagents were of analytical grade and purchased by Sigma-Aldrich (Milan); all solutions were prepared with deionized water. nZVI were characterized by Dynamic Light Scattering method, using a Brookhaven Plus 90 equipment. Particle Size Distribution (PSD) and Zeta potential (Z, mV) were determined, varying the nZVI solution pH in the range 6–10 and measured by a Crison 421 pH-meter. Fresh-prepared and used nZVI were also characterized by XRD (BRUKER D8 ADVANCE). TEM, DLs and Zeta Potential characteristics were previously described in studies of Vilardi *et al*.^17,41^. A synthetic solution of K_2_Cr_2_O_7_ was prepared by dissolving the reagent in deionized water and the different concentrations were achieved by providing a dilution.

### Kinetic study

The synthetized nanoparticles have been tested in Cr(VI) solutions at different R = nZVI/Cr(VI) molar ratios (0.5, 1, 1.5, 2) considering the redox reaction reported below:

1 $$Fe(0)+Cr{O}_{4}^{2-}+4{H}_{2}O\to Fe{(OH)}_{3}+Cr{(OH)}_{3}+2O{H}^{-}$$

kinetic data were fitted to a pseudo-n-th order kinetic model as described by Bavasso *et al*.^11^ and expressed by the following equation:

2 $$Cr{(VI)}^{n-1}=\frac{Cr{(VI)}_{0}^{n-1}}{1+(n-1)Cr{(VI)}_{0}^{n-1}kt}$$

where n is the reaction order and k (mg^1−n^/l^1−n^ s) is the kinetic constant. The non-linear regression of experimental data was accomplished in Excel environment, using the non-linear solver of Excel (Microsoft). The experiments were conducted in borosilicate beaker of 100 ml of volume placed on an orbital shaker, fixing the initial concentration of Cr(VI) equal to 52 mg/l (i.e. 1 mM) and at selected initial concentration of nZVI (28, 56, 84 and 112 mg/l, i.e. 0.5, 1, 1.5 and 2 mM, respectively) to investigate the influence of nZVI dosage on Cr(VI) removal process performances. A stock solution of K_2_Cr_2_O_7_ 0.1 M was used as a source of Cr(VI). The mixing intensity was fixed at 500 rpm, basing on preliminary tests, whereas the temperature was kept constant at 25 °C through a water bath. At selected time interval (0, 10, 20, 30, 40, 50, 60, 120, 180, 300, 600 and 900 s) a liquid sample was taken and filtered by a syringe filter (100 nm, Nylon, Cameo): the residual Cr(VI) was measured by diphenylcarbazide method, as reported in a previous work^17^.

### Preparation of seeds

The experiments were performed under laboratory conditions using tomato seeds (*L. esculentum* var. F1 Zucchero) purchased from the company Magnani sementi s.r.l (Italy). The seeds were surface sterilized with 10% sodium hypochlorite solution for 10 min, then vigorously rinsed with sterilized deionized water before transferring into Petri dishes with a double layer of filter paper.

### Preparation of Cr(VI) solution

A stock solution of Cr(VI) 0.1 M was prepared using an accurately weighed quantity of the potassium dichromate (K_2_Cr_2_O_7_, Merck) in deionized water. Experimental solutions of the desired concentrations were obtained by successive dilutions. A volume of 4 ml of Cr(VI) contaminated water was added to Petri dish. To prepare the Cr(VI) + nZVI solution, a stechiometric dosage of nZVI and Cr(VI) has been used (see kinetic study in materials and methods).

### Tomato seed germination

Tomato seeds (fifty per treatment) were placed on petri dishes (90 mm in diameter) with sheets of sterilized filter papers and maintained in growth chambers at 24 °C in continuous darkness condition. To evaluate the biological effect of nZVI and Cr(VI) on seed germination, an amount of 4 ml of nZVI or Cr(VI) was applied at different concentrations as follows: deionized water (control), Cr(VI) 5 mg L^−1^, nZVI 5 mg L^−1^, Cr(VI) + nZVI 5 mg L^−1^, Cr(VI) 50 mg L^−1^, nZVI 50 mg L^−1^, Cr(VI) + nZVI 50 mg L^−1^, Cr(VI) 100 mg L^−1^, nZVI 100 mg L^−1^, Cr(VI) + nZVI 100 mg L^−1^, Cr(VI) 1000 mg L^−1^, nZVI 1000 mg L^−1^, Cr(VI) + nZVI 1000 mg L^−1^. The seeds were incubated at 24 °C and 70% relative humidity under a 16 h/8 h light/dark cycle (approximately 130 µmol m^−2^ s^−1^). The number of seeds germinated was counted at 3, 6 and 10 days and expressed as seed germination percentage. Seeds were considered as germinated when their radicle showed at least 2-mm length. Each Petri dish was treated as one replicate and all the treatments were repeated five times.

### Evaluation of the effects of Cr(VI) and nZVI on tomato seedlings

To establish the minimum Cr(VI) concentration able to adversely affect the plant growth, tomato seeds were treated with Cr(VI) contaminated water as follows: deionized water (CTRL), contaminated water with Cr(VI) at 1.5, 3, 5, 12.5, 25, 50, 100, 250, 500 and 1000 mg ^L−1^. A volume of 4 ml of Cr(VI) contaminated water was added to each Petri dish. After 7 days of treatment the length of hypocotyl and root were measured using the ImageJ software as described by Schneider *et al*.^42^.

Since, Cr(VI) 5 mg L^−1^ resulted the minimum concentration which significantly reduced the hypocotyl and root length, the ability of nZVI 5 mg L^−1^ to remediate Cr(VI) contaminated water was evaluated. Tomato seeds were treated as follows: deionized water (CTRL), Cr(VI) 5 mg L^−1^, nZVI 5 mg L^−1^ and Cr(VI) + nZVI 5 mg L^−1^. After 7 days of treatment the length of hypocotyl and root were measured.

### Evaluation of the effects of the treatment with only nZVI on tomato plant performance

Tomato seeds were germinated and plants grown in different size of pots on universal substrate (COMPO SANA®, Germany), at 24 °C and 70% relative humidity, under a 16 h/8 h light/dark cycle (approximately 130 µmol m^−2^ s^−1^). Plants were daily treated with nZVI 5 mg L^−1^ until to the full ripening. Three pot sizes were used: 1) pots of 0.10 L were used for plant germination up to 15 days after germination and a volume of 5 ml of nZVI solution was added; 2) pots of 0.50 L were used from 15 to 60 days after germination and a volume of 50 ml of nZVI solution was added; 3) pots of 1.7 L were used from 60 to 180 days after germination and a volume of 150 ml of solution was added. Controls were treated with the corresponding water volumes. After 35 and 64 days of growth the following parameters were evaluated: plant height, number of nods and internodes, leaf chlorophyll and carotenoid content. The number of flowers per inflorescence and fruits were evaluated at 65 days of plant growth. Fresh tomato fruits were harvested at the same stage of ripening, in order to evaluate their carotenoid, fat-soluble vitamin and nicotianamine content by means of high-performance liquid chromatography - tandem mass spectrometry (HPLC-MS/MS).

### Determination of total carotenoid and chlorophyll content in the leaves

To evaluate the carotenoid and chlorophyll content in the leaves, samples were weighted and added to 96% methanol (v/v), with 1:50 ratio. The samples were maintained at 4 °C in dark conditions for 72 h, and then the supernatant was separated and analyzed by a Shimadzu UV-1280 spectrophotometer. Chlorophylls a and b were read at 662 and 646 nm, respectively, and total carotenoids at 470 nm^43^. Quantification was carried out according to the formulas of Lichtentaler and Wellburn^44^.

### Analysis of carotenoids and fat-soluble vitamins by HPLC-MS/MS with atmospheric pressure chemical ionization (APCI) in mature fruits

Carotenoids and fat-soluble vitamins were determined as described by Gentili *et al*.^45^. Briefly, a pool of fruits was homogenized (Ultra Turrax homogenizer, Janke & Kunkle, Staufenim Breisgau, Germany) and 4-g aliquots of were transferred into polypropylene centrifuge tubes (50-mL capacity). Magnesium carbonate and BHT were both added in a ratio of 10:1, w/w. The analytes were extracted by means of alkaline hydrolysis at 25 °C for 15 h under magnetic stirring. To this end, each aliquot was treated with 6 mL of absolute ethanol, containing 0.1% (w/v) BHT, and 1 mL of 50% (w/v) aqueous KOH. The digest was diluted with 3 mL of water and the analytes extracted with 4 mL of hexane, containing 0.1% (w/v) BHT, stirring for 5 min, vortexing for 5 min and centrifuging at 6000 rpm at 4 °C for 10 min. The extraction was repeated six times. Thereafter, the collected hexane fractions were washed twice with 8-mL aliquots of water. The extract was transferred into a glass tube and concentrated up to 100 µL, evaporating at 30 °C under a N_2_ flow. A 200-µL final volume was obtained by adding a 2-propanol:hexane (75:25, v/v) solution containing 0.1% (w/v) BHT. Finally, 40 µL was injected into the HPLC-APCI-MS/MS system.

Analytes were separated on a ProntoSIL C30 column (4.6 × 250 mm, 3 μm) (Bischoff Chromatography, Leonberg, Germany), preceded by a guard C30 column (4.0 × 10 mm, 5 μm), chilled at 19 °C. The elution was carried out under non aqueous-reversed phase (NARP) conditions, using methanol (phase A) and 2-propanol/hexane (50:50, v/v; phase B); the applied gradient was as follows: t_0−1 min_, 0% B; t_1−15 min_, 0−75% B; t_15−15.1 min_, 75−99.5% B; and t_15.1−30.1 min_, 99.5% B. The mobile phase at a flow rate of 1 mL/min and was completely introduced into the APCI source of the MS detector.

Analytes were detected with an APCI source operating in positive ion mode. The working MS parameters were the following ones: the needle current at 3 μA; the probe temperature at 450 °C; the curtain gas was high-purity nitrogen at 40 psi; the collision gas was nitrogen at 4 mTorr; the nebulizer gas was air at 55 psi; the makeup gas was air at 30 psi.

### Nicotianamine analysis by HPLC-MS/MS with electrospray ionization (ESI) in ripe fruits

Nicotianamine was extracted from 1 g of homogenized fresh tomato by adding 10 mL of water. After stirring and centrifugation for 10 min (6000 rpm, 25 °C), the supernatant was filtered (0.45 μm hydrophilic PTFE filter from Millipore) and diluted with a 0.1% (v/v) formic acid aqueous solution by a factor of 25. A 5-µL volume of the final extract was injected into the HPLC-ESI-MS/MS system. Chromatography was performed using a C18 XTerra column (150 × 2.1 mm, 3.5 µm from Waters) kept at 35 °C. Mobile phases consisted water (phase A) and water/acetonitrile (30:70) (phase B), both of them containing 0.1% (v/v) formic acid. The flow rate of the mobile phase was 140 µL/min and it was entirely introduced into the mass spectrometer. The applied gradient was: t_0−0.5 min_, 100% A; t_0.5−15 min_, 100% B.

The ESI detection was performed in positive ionization. The working MS parameters were the following ones: the capillary voltage at 5500 V; the probe temperature at 450 °C; the curtain gas was high-purity nitrogen at 5 L/min; the collision gas high-purity nitrogen at 4 mTorr; the nebulizer gas was air at 2 L/min; the drying gas was air at 20 L/min.

### Quantitative analysis of carotenoids, fat-soluble vitamins and nicotianamine

Carotenoids, fat-soluble vitamins and nicotianamine were quantified by external calibration. Calibrators were prepared so that the interpolated concentrations of the real samples fell into the linear dynamic range. Linear regression was calculated with Microsoft Excel 2010, obtaining r^2^ coefficients as high as 0.999 for nicotianamine and greater than 0.970 for the fat-soluble micronutrients. The analyte concentrations in tomato fruits were calculated from the straight-line equation, taking into account the dilution factor (nicotianamine), as well as the final volume of the extracts and the weight of fruit samples.

### Determination of iron level in leaves, fruits, roots and soil

Iron (Fe) concentration after treatment with nZVI (5 mg L^−1^) was determined after acid digestion of lyophilized samples according to the EPA 3050B method. Pooled solid samples obtained from each plant organ and soil were previously milled, and the organics were removed by drying the powder at 550 °C–600 °C for 3–4 h. The analysis of Fe concentration in the samples was performed by absorption spectrophotometry (FAAS) analysis (Agilent AA DUO 240 Fs instrument).

### Statistical analysis

Data were statistically analyzed using Sigma Plot (version 12.0, Sysstat Software, Inc., USA). The results were reported as mean ± standard error. Differences between groups were tested using t-test for chlorophyll, carotenoids, vitamins and nicotianamine levels. One-way ANOVA followed by Holm-Sidak Test was used to assess differences among nZVI and Cr(VI) treatments for root and hypocotyl length. Chi-Square test was used to analyze experimental data on the seed germination. A *P* < 0.05 value was accepted as statistically significant.

## Supplementary information

Supplementary info.
