# Supplementary material for: Remediation of hexavalent chromium contaminated water through zero-valent iron nanoparticles and effects on tomato plant growth performance
Source: Sci Rep. 2020 Feb 5;10:1920. doi: 10.1038/s41598-020-58639-7 (PMC7002744; doi:10.1038/s41598-020-58639-7)
Supplement: Supplementary file 1 — Supplementary info. [file 41598_2020_58639_MOESM1_ESM.pdf]

Supplementary information

Remediation of hexavalent chromium contaminated water through zero-valent iron nanoparticles and effects on tomato plant growth performance

Elisa Brasili[1+], Irene Bavasso[2+], Valerio Petruccelli[1], Giorgio Vilardi[2], Alessio Valletta[1], Chiara Dal Bosco[3], Alessandra Gentili[3], Gabriella Pasqua[1*], Luca Di Palma[2]

[1]Sapienza University of Rome, Department of Environmental Biology, Rome, 000185, Italy
[2]Sapienza University of Rome, Department of Chemical Engineering Materials Environment, Rome, 00185, Italy
[3]Sapienza University of Rome, Department of Chemistry, Rome, 00185, Italy

[*]Corresponding author:
gabriella.pasqua@uniroma1.it;
+these authors contributed equally to the work

Table S1. LC-MRM parameters used for the quantitative analysis of fat-soluble micronutrients and nicotianamine in tomato fruits

**Analytes**	**Retention time[a] Average± SD (min)**	**MRM transitions[b] (m/z)**	**Ion ratio (%)[c] Average± SD**
β+γ-tocopherol	8.5 ± 0.3	416.3/191.2<br>416.3/151.1	20 ± 5
α-tocopherol	9.2 ± 0.4	430.2/205.1<br>430.2/165.1	9 ± 6
all-*trans*-lutein	9.8 ± 0.4	551.5/175.0<br>551.5/135.2	68 ± 9
all-*trans*-zeaxanthin	10.5 ± 0.6	569.6/175.2<br>569.6/119.0	51 ± 8
all-*trans*-phytoene	15.2 ± 0.4	545.5/69.0<br>545.5/81.0	74 ± 9
all-*trans*-phytofluene	15.5 ± 0.4	543.4/69.0<br>543.4/81.0	80 ± 10
all-*trans*-β-carotene	16.8 ± 0.5	537.5/119.1<br>537.5/177.2	75 ± 9
all-*trans*-ζ-carotene	17.5 ± 0.5	541.7/69.0<br>541.7/81.0	86 ± 10
all-*trans*-γ-carotene	19.3 ± 0.5	537.5/119.1<br>537.5/177.2	95 ± 10
all-*trans*-lycopene	25.8 ± 0.6	537.2/177.0<br>537.2/119.0	40 ± 8
5-*cis*-lycopene	26.4 ± 0.6	537.2/177.0<br>537.2/119.0	48 ± 9
nicotianamine	2.73 ± 0.5	304.3/185.2<br>304.3/286.3	80 ± 6

[a]The retention times are reported as arithmetic average of ten replicates. [b] The first line reports the least intense MRM transition (qualifier, q) and the second line the most intense one (quantifier, Q).[c] The ion ratio (relative abundance) between the two MRM transitions is calculated as percentage intensity ratio of I_q/I_Q; the results are reported as arithmetic average of ten replicates.

Table S2. Linear regression parameters used for the quantitative analysis of fat-soluble micronutrients and nicotianamine in tomato fruits

**Analyte**	**Calibration curve**	r^2
β+γ-tocopherol	y = 4.5961x + 5068.4	0.979
α-tocopherol	y = 16.761x + 24279	0.991
all-*trans*-lutein	y = 10.644x + 22.139	0.995
all-*trans*-zeaxanthin	y = 0.749x - 56.333	0.999
all-*trans*-phytoene	y = 0.4603x + 9.7887	0.999
all-*trans*-phytofluene	y = 0.8187x + 12.241	0.990
all-*trans*-β-carotene	y = 1.9475x - 3.8153	0.998
all-*trans*-ζ-carotene	y = 3.6421x - 59.037	0.997
all-*trans*-γ-carotene	y = 1.1872x - 0.3783	0.971
all-*trans*-lycopene	y = 0.1043x + 1.7143	0.989
nicotianamine	y = 4599.1x + 326.86	0.999
